# DEFHAZ: A Mechanistic Weather-Driven Predictive Model for *Diaporthe eres* Infection and Defective Hazelnut Outbreaks

**DOI:** 10.3390/plants11243553

**Published:** 2022-12-16

**Authors:** Marco Camardo Leggieri, Roberta Arciuolo, Giorgio Chiusa, Giuseppe Castello, Nicola Spigolon, Paola Battilani

**Affiliations:** 1 Department of Sustainable Crop Production, Università Cattolica del Sacro Cuore, 29121 Piacenza, PC, Italy; 2 Soremartec Italia S.r.l., Piazzale Pietro Ferrero 1, 12051 Alba, CN, Italy

**Keywords:** *Corylus avellana* L., rotten hazelnut, system analysis, predictive model, meteorological data, fungi, *Phomopsis*

## Abstract

The browning of the internal tissues of hazelnut kernels, which are visible when the nuts are cut in half, as well as the discolouration and brown spots on the kernel surface, are important defects that are mainly attributed to *Diaporthe eres*. The knowledge regarding the *Diaporthe eres* infection cycle and its interaction with hazelnut crops is incomplete. Nevertheless, we developed a mechanistic model called DEFHAZ. We considered georeferenced data on the occurrence of hazelnut defects from 2013 to 2020 from orchards in the Caucasus region and Turkey, supported by meteorological data, to run and validate the model. The predictive model inputs are the hourly meteorological data (air temperature, relative humidity, and rainfall), and the model output is the cumulative index (*Dh-I*), which we computed daily during the growing season till ripening/harvest time. We established the probability function, with a threshold of 1% of defective hazelnuts, to define the defect occurrence risk. We compared the predictions at early and full ripening with the observed data at the corresponding crop growth stages. In addition, we compared the predictions at early ripening with the defects observed at full ripening. Overall, the correct predictions were >80%, with <16% false negatives, which confirmed the model accuracy in predicting hazelnut defects, even in advance of the harvest. The DEFHAZ model could become a valuable support for hazelnut stakeholders.

## 1. Introduction

Hazelnut (*Corylus avellana* L.), which is cultivated worldwide in areas with mild climates and high humidity, has a global production higher than 850,000 tonnes, according to the FAOSTAT [1]. Defects such as blemishes, areas of discolouration or stains in marked contrast with the rest of the hazelnut, and internal browning observed after half-cutting dramatically affect the commercial quality, yield, and market value [2]. Defective hazelnuts that do not comply with the quality standards required by the market [3] represent a critical issue for producers.

In recent years, researchers have reported the fungi of the genus *Diaporthe* as the principal causative agent of hazelnut kernel defects, including the brown spots on the surface (visible defects) and those visible after hazelnut half-cutting (hidden or internal defects). Based on the molecular characterisation of a *Diaporthe* population, researchers identified the *D. eres* species complex as the main factor [4]. The first study area was in Chitatskari (Georgia) in the Caucasus [5]; however, the researchers confirmed the results in Turkey [4,6].

Researchers have primarily studied *Diaporthe* spp., and their asexual, more frequently observed stage *Phomopsis* spp., both referred to as *Diaporthe* after the recommendation of Rossman et al. [7], on grapes. They have only described the infection cycle of *Diaporthe* spp. on citrus (*D. citri*) and grapevine (*D. ampelina*), with the inclusion of the sexual stage in the former. *Diaporthe* spp. are monocyclic pathogens, as confirmed for *P. viticola/D. ampelina* [8], with only one infection cycle for each growing season of the host crop. Therefore, the disease incidence and severity depend on the primary inoculum, which comes from pycnidial conidiomata that overwinter during the inactive period of the plant on infected plant parts or crop debris. At maturation, pycnidial conidiomata produce cirrhi, which contain conidia [9]. Conidia are dispersed by rain and insects for *D. eres* [10], or by heavy rain for *D. ampelina* [11], which the authors studied on apple trees and grapevine, respectively. Conidia dispersal enables the beginning of the infection cycle, with contact between the fungus and host plant. *D. ampelina* conidia germinate at temperatures between 5 and 35 °C [12,13], and during wet periods with >5 h of rain with high RH [14]. The growing mycelium penetration occurs through the stomata, epidermis, and lenticels for *D. eres* in apples [12], and through the stomata or wounds for *D. ampelina* [8]. No data are available regarding hazelnut.

Researchers have primarily described the disease symptoms for grapevine. The disease occurs, initially superficially, on young branches, and more rarely on the trunk and thick branches [13], and it can also affect the leaves and fruits. However, even if the infection occurs in the field, symptoms are visible during fruit storage [10,12,13] and therefore after long periods of latency.

Recently, other researchers have confirmed the relevance of *Diaporthe* spp., and mainly *D. eres*, in hazelnut. The authors of [15] associated *D. eres* with trunk cankers in Oregon, Guerrero-Contreras et al. [16] cited *D. foeniculina* as the cause of the black tips and necrotic spots on hazelnut kernels in Chile, and the authors of [17] detected *D. rudis* in hazelnut kernels with visible mould in Oregon. Similarly, researchers have associated multiple species of *Diaporthe* with grapes with the prevalence of *D. ampelina* [11]. Until now, the focus of the research has been on the causal agents of defective hazelnuts, and scholars have recently published interesting ecological studies [18]; however, suggestions for their management in the field are still lacking in terms of the preventive actions, effective active ingredients, and proper scheduling of interventions. The rational control of harmful organisms on crops is fundamental to ensuring their agricultural productivity, farmers’ profits, and economic and environmental sustainability. In this context, researchers are increasingly adopting predictive models as crucial supporting tools [19], which are considered the core of IPM, according to the EU regulation (Directive 2009/128/EC) [20]. The models can be used to predict disease outbreaks and development on crops during the growing season until harvesting, and they rationally support decisions related to disease management (e.g., fungicide applications). 

Pscheidt and Heckert took the first tentative step towards the development of an empirical model to predict hazelnut kernel mould that is not related to a specific fungal species [21]. They collected data on two susceptible breeding selections and two commercial cultivars, and they observed the kernel mould development over time, concluding that the initial disease incidence is an effective predictor of the area under the disease progress curve (AUDPC). In a more recent paper, the authors present a model to predict rotten hazelnut kernels; in this case, they did not focus on a specific causal agent but included the *Diaporthe* spp. [22]. The authors confirmed the strict dependence of rotten hazelnuts on the precipitation amount and timing, and on the role of the plant susceptibility, and they suggest the effects of other environmental and agronomic factors on the rot incidence that they did not include in the model. A mechanistic model for *D. eres*, based on the infection cycle of the fungus on hazelnut, is not yet available but would be beneficial.

Mechanistic predictive models are more accurate and robust than empirical ones [23,24]. We can conceptually develop mechanistic predictive models by using system analysis [25], by mathematically taking advantage of published data [26], or by using data obtained by fitting for experiment purposes. Recently, researchers combined machine learning with a mechanistic model to account for the role of the cropping system in the mycotoxin occurrence in maize, obtaining substantial improvements in the model accuracy [27]. The application of machine learning (ML) to farm management systems is quickly evolving and cannot be neglected, as it provides richer recommendations and insights for subsequent decisions and timely actions [28].

In the present study, we aimed to develop the first weather-based mechanistic model to predict the epidemics and lifecycle of *D. eres* in hazelnuts, as well as the occurrence of the visible and hidden symptoms in hazelnut kernels. For this purpose, (i) we retrieved information from the literature on the pathosystem, (ii) we drew a conceptual model based on system analysis principles, (iii) we developed an algorithm to both quantitatively and dynamically simulate the system, (iv) we validated the model, and (v) we identified and discussed the gaps in the knowledge that require further research.

## 2. Results

### 2.1. System Analysis of D. eres Lifecycle

**Figure 1:**
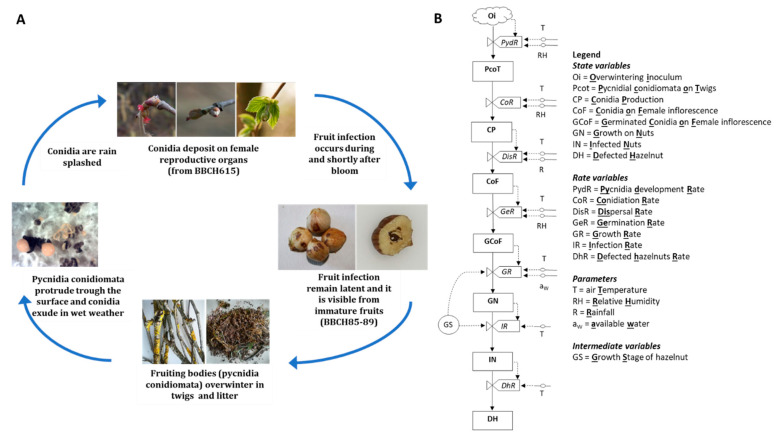
(**A**) Simplified description of *Diaporthe eres* infection cycle on hazelnut, and (**B**) relation diagram. Boxes are state variables; solid arrows represent fluxes and directions of states; dotted arrows represent fluxes and directions of variables (data input); circles symbolise intermediate variables; “bow ties” are valves in fluxes (rates) (we report the acronym explanations directly in the figure).

We present the relational diagram of the model in Figure 1. The first state variable of the model consists of the overwintered inoculum (Oi). The T and RH trigger the pycnidia development (PydR) on twigs (PcoT), as well as the conidia production (CoR). The produced conidia (CP) are then dispersed, and the T and RH regulate the dispersal rate (DisR). Conidia can land on female hazelnut inflorescence (SoF), which is the third state variable of the model. Driven by the germination rate (GeR) under the influence of the T and RH, conidia can germinate (GCoF). When hazelnuts are in the suitable growth stage (GS), germinated conidia can grow on the nuts (GN), and the *a*_*w*_ and T act as the driving variables in the prediction of the growth rate (GR). Hazelnuts become infected (IN), and the T regulates the infection rate (IR). Finally, through the defective hazelnut rate (DhR), infected hazelnuts may become defective (DH) under the T influence. The model runs from 1 January (Julian date), with a time step of 1 h.

### 2.2. Algorithm and Model Development

#### 2.2.1. Overwintering Inoculum (Oi), Pycnidia Production Rate (PydR), and Conidiation Rate (CoR)

Conidial production depends on the fungal inoculum (Oi) amount and the ecological conditions; the Oi is naturally available in hazelnut orchards. Arciuolo et al. [18] computed the data on the PydR as a function of the growing degree days (GDDs) for both the temperature (*T*) and water activity (*a_w_*); the base for the GDDs was equal to 0 °C. The function used to fit the data was a logistic equation, which is written as follows:

(1) YT/aw=c(1+exp(a+b∗x))

where *a*, *b*, and *c* are the estimated parameters, and *x* is the independent variable. We present the estimated parameters in Table 1. The adjuster *R*^2^ values were 0.950 and 0.972 for the *T* and *a_w_* functions, respectively.

Based on unpublished data [29], we fixed the GDDs as ≥788 and the rainfall as >0.2 mm as the thresholds for conidiation occurrence. Therefore, we do not describe the CoR according to a function but based on a yes/no answer.

#### 2.2.2. Dispersal Rate (DisR)

No experimental data were available in the literature regarding *D. eres* dispersal. Based on general knowledge of the ecology of the *Phomopsis*/*Diaporthe* spp., conidia are dispersed by rain and insects [10,12]. The model assumes that conidia are also dispersed when the weather conditions are suitable for sporulation, which are days with R > 0.2 mm.

#### 2.2.3. Germination Rate (GeR)

Arciuolo et al. [18] computed the data on the *D. eres* germination (GeR) as a function of the T using the Bete equation (Equations (2) and (3), Analytis [30]), and they computed the data on the RH using a polynomial equation (Equation (5)). We present the estimated parameters for Equations (2) and (3) in Table 1. The adjusted *R*^2^ values for the fitted *D. eres* germination data were 0.793 and 0.975, respectively, for the *T* and *RH* equations (Table 1). The standard parameter errors were lower than the parameter values, confirming the goodness of the fit of the applied equations.

(2) $$Y\left(T\right)={\left(a\times {\left(Teq\right)}^{b}\times \left(1-Teq\right)\right)}^{c}$$

where *Teq* is the equivalent of the *T*, computed as:

(3) Teq=(T−Tmin)(Tmax−Tmin)

(4) $$Y\;\left(RH\right)=a\times RH^{2}+b\times RH+c$$

#### 2.2.4. Infection Rate (IR)

No data are available in the literature regarding *D. eres* infection, and artificial inoculation trials, managed until now, are insufficient to define what happens after conidia germination. For the model development, we assumed that the flowering period was suitable for the hazelnut infection by the fungus, which we improved in the model refinement (see Section 2.3 for details).

#### 2.2.5. Growth Rate (GR)

The *D. eres* GR on hazelnuts depends on the *T* and *a_w_*, and we obtained the quantitative data on the colony growth from the literature [18]. Researchers have observed fungal growth in T ranges of 5–40 °C, and the optimum was reached at around 20–25 °C. Growth has not been observed for *a_w_* < 0.87, and the highest GR was reached for *a_w_* > 0.97. We modelled the GR using a Bete equation as a function of *T* (Equations (2) and (3), and Table 1 for equation parameters), and a logistic equation for *a_w_* (Equation (1), and Table 1 for parameters). The adjusted *R*^2^ values were acceptable for both factors (≥0.96, Table 1). The standard parameter errors were lower than the parameter values, confirming the effectiveness of the applied equations.

#### 2.2.6. Defective Hazelnut Rate (DhR)

Defective hazelnuts (Dh), which are the last step in the relational diagram, are the result of the infection cycle. We computed the Dh cumulative index (*Dh-I*) following the described steps and using georeferenced meteorological data from 1 January to BBCH81 (when from 10% to 50% of the hazelnut shells changed colour [31]) and BBCH89 (when the nuts separated from the husks at the basal scar, the basal scar turned brown, and the nuts fell to the ground [31]).

### 2.3. Model Refinement

#### Crop Susceptibility to Infection

We linearly regressed all the indexes generated by the developed model (independent variables: *Dh-I 30_04*; *Dh-I 30_05*; *Dh-I 30_06*; *Dh-I NL*) using a stepwise approach versus the observed data on the hazelnut defects (dependent variables). We obtained the best F-test result using the *Dh-I* obtained assuming crop susceptibility until the end of May (*Dh-I 30_05*; F = 0.00 versus F > 0.3).

Therefore, we collected the model output (*Dh-I 30_05*) at both hazelnut growth stages (BBCH81 and BBCH89) using the reference Julian data provided in the database.

**Figure 2:**
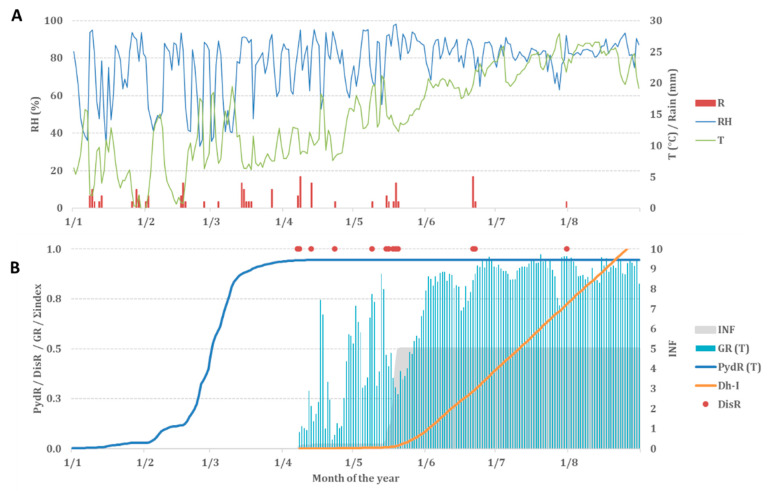
Example of DEFHAZ input and output in Georgia, 2016. (**A**) Weather data as input: air temperature (T) (green line in °C), rainfall (R) (red bars in mm), and relative humidity (RH) (blue line in %). (**B**) Trends of main model output: blue line (PydR (T)) is daily production rate of pycnidia, red dots (DisR) represent days when inoculum was dispersed, light blue bars (GR(T)) are *D. eres* growth rates, grey area is (INF) cumulative infection, and orange line (*Dh-I*) is cumulative infection index.

We present an example of the DEFHAZ model input (Figure 2A) and output (Figure 2B) run in 2016 in Chitatskari, Georgia, in Figure 2.

### 2.4. Probability Function

#### Binary Logistic Regressions for HD and TD Estimations in Hazelnuts: Internal Validation

Based on the field data collected, the amounts of hazelnuts with HDs and TDs above the considered threshold (≥1% of incidence) for the BBCH81 growth stage were 4.8% and 12%, respectively; these values increased for the BBCH89 growth stage, with 16% and 38% of the samples above the HD and TD thresholds, respectively.

We developed the binary logistic regressions (Equation (1)) using the HD or TD incidence of the samples as the dependent variable at a threshold of 1% incidence, and we used *Dh-I 30_05*, generated as the output by the predictive model, as the independent variable. We based these on the 44 orchards previously mentioned (Table 2).

### 2.5. Model Internal Validation

We present the results of the hazelnut model validation in the contingency matrix (Table 3), expressed as the model’s capability of predicting the observed incidences of HDs and TDs. We tested this capability in different potential scenarios according to the crop phenology. The growth stage scenarios were as follows: (i) BBCH81 vs. BBCH81 (model output calculated in BBCH81 and compared with the observed HD/TD incidences in BBCH81); (ii) BBCH89 vs. BBCH89 (model output calculated in BBCH89 and compared with the observed HD/TD incidences in BBCH89); (iii) BBCH81 vs. BBCH89 (model output calculated in BBCH81 and compared with the observed HD/TD incidences in BBCH89).

We obtained the model accuracy by the sum of the true positive (TP) and true negative (TN) results, which ranged from 71% to 95%. The model could not correctly predict the TP samples in all the considered growth stages for the HD incidence; however, the number of samples observed with an HD defect incidence higher than 1%, which was the fixed threshold to develop the logistic equations, was extremely low. Therefore, the errors accounted for by the developed equations for the HDs were only underestimations or false negatives (FNs). Regarding the TD hazelnuts, the logistic equations had accuracies between 71% and 89%. We observed the highest FN amount for the BBCH81 vs. BBCH89 scenario, and the highest FP for the BBCH89 vs. BBCH89 scenario, with 5%.

## 3. Discussion

Hazelnut defects cause yield losses because of the noncompliance with the quality standards required by the market [5]; therefore, the industry increasingly looks for high-quality in-shelled fruits [32]. Quality defects often affect hazelnut fruits, and they are possibly associated with off-flavours [17]. Recently, Battilani et al. [5] identified *Diaporthe* spp. as candidate etiological pathogens in the Caucasian region, as did Arciuolo et al. [4,6] in Turkey, highlighting the primary role of *D. eres*. In agreement with the European vision of integrated pest management (IPM) stressed by the Green Deal, predictive models have attracted increasing interest for the prediction of disease epidemics during crop-growing seasons as support for rational disease management and the mitigation of the disease severity at harvest.

In this study, we developed a weather-driven pathogen-focused mechanistic model for *D. eres* epidemics in hazelnut to predict defective hazelnut outbreaks [4]. We developed the model, called DEHHAZ, based on well-documented data and an understanding of the disease; nevertheless, due to knowledge gaps, we made some assumptions to complete the model. Gonzalez-Dominguez et al. [11] followed the same modelling approach considering *D. ampeliana*, which is the causal agent of the *Phomopsis* cane and leaf spot of grapevines (known in Europe as “excoriose”), for which assumptions were also required for the pathosystem, confirming the incomplete information commonly available for the *Diaporthe* spp.

The DEFHAZ model uses hourly data on the T, RH, and R collected from 1 January as the input, and it produces the *Dh-I* as the daily output, which is the cumulative infection index used to predict the probability of the defective hazelnut occurrence above a 1% incidence for both hidden defects (HDs) and total defects (TDs). We managed the model validation by comparing the observed defect incidences [4,5] with the model predictions. We obtained the best DEFHAZ performance (acceptable accuracy, with 95% correct predictions) for the HDs in the BBCH81 growth stage, which is a satisfactory result but does not refer to the growing season’s end. The DEFHAZ model better predicted defective hazelnuts in the BBCH81 growth stage than in the BBCH89 growth stage, both for HDs and TDs, which is not surprising due to the underlined lack of knowledge regarding the length of the incubation period, which is intended to be time-elapsed from the fungal infection to the symptom outbreak, which limits the predictive capacity of the model during the ripening period, when the visible defective hazelnuts increase. Nevertheless, the model prediction run at the BBCH81 growth stage predicted the defect occurrences at harvest (BBCH89): the correct predictions were 84% for the HDs and 71% for the TDs, which makes the model useful in practice to support stakeholder decisions. In fact, we can reasonably predict the quality of the hazelnut production, depending on the production area, with a large advance compared with the harvest.

We tested the model performances on 44 orchards, which resulted in different “year” × “location” combinations. The dataset is comprehensive, and it makes the model validation robust. The extension of the validation by including more orchards and eventually additional areas and years is desirable to consolidate the obtained results.

We based this model on weather, and we did not include any other factors. Based on the results reported by Pscheidt and Heckert [21] and Valeriano et al. [22], other factors are relevant to enhancing defective hazelnuts, such as the variety susceptibility to fungal infection. Therefore, the prediction capacity of the DEFHAZ model is adequate; however, it could be improved by including other variables. The combination of the DEFHAZ model with a machine learning approach, when additional knowledge is available, including the roles of the hazelnut variety and cropping system in the defect occurrence, will be crucial to the development of a decision support system that is in line with so-called “knowledge-based agriculture” [27].

Furthermore, we made several assumptions during the development of the DEFHAZ model due to a lack of knowledge. The first assumption of our model concerns the role of the meteorological parameters in the process of cirrhus production, which is possible when rainfall occurs, based on the experiences of *D. eres* on apple [33] and *D. ampelina* on grape [11]. Another weak point is conidia germination. Data were available, but only under a narrow range of RH conditions (94%, 97%, and 100% RHs; [18]), We need more detailed experiments to better describe the conidia germination potential with different RH values, as well as time regimes. In a recently developed model already mentioned [22], the authors neglected this aspect and assumed that the dispersed conidia were suitable for the infection under the proper combination of leaf wetness and temperature, which could have had a strong negative impact on the model performances.

In terms of the crop susceptibility windows, we established the DEFHAZ model to have possible infection until the end of May, which we based on the regression analysis using a stepwise approach, and which is in partial agreement with Valeriano et al. [22], who considered hazelnut to be most susceptible to infections during female flowering, and therefore over a narrower period. To confirm the reasonable period for primary infection, we require studies with the artificial inoculation of *D. eres* α conidia in planta, and possibly with the subsequent incubation at different temperatures.

The latent period length, from fungal infection to visible symptoms, is another crucial unknown aspect that we did not include in the model. Gonzalez-Dominguez et al. [11] highlighted the same lack of knowledge for *D. ampelina* in grapes. The correct estimation of the latent period for a monocyclic disease, such as the defective hazelnut considered in this study, together with the crop susceptibility window, are the key components in defining the possible symptoms of an outbreak, and they merit research efforts to improve the model predictions. Arciuolo et al. [18] argue that the *a*_*w*_ status of hazelnut could play a role, as several steps of the *D. eres* infection cycle are strictly dependent on the *a*_*w*_ in the kernels, from fruit set to harvest. In the future, the scientific community should address this issue, which would be beneficial to improving the model accuracy.

We cannot neglect the fungal community that co-occurs with *Diaporthe* spp., as researchers have reported an incidence greater than 10% for fungi isolated from defective hazelnuts in Turkey from the *Botryosphaeria*, *Fusarium*, *Aspergillus*, and *Penicillium* species [6]. Other authors have reported co-occurring fungi in hazelnuts in different geographic areas [17,34,35]. Co-occurring microorganisms of other pathosystems are catching the attention of scientists [36,37], and they have acquired increasing relevance due to climate change for mycotoxin-producing fungi [38,39,40]. In particular, the co-occurrence of *Diaporthe* with the *Aspergillus* section *Flavi* has safety relevance due to the large proportion of nuts that fail quality standards for human consumption, and especially in Europe [41,42,43]. Therefore, we need to elucidate this complex aspect in perspective to obtain a higher predictive accuracy for the DEFHAZ model.

In conclusion, the DEFHAZ model has the potential to support farmers and buyers in their assessment of the risk areas for hazelnut defects, accounting for both the hidden and total defects, and it can be performed at early ripening, around one month in advance of harvest time. Furthermore, the estimation of the crop susceptibility to infection provides essential insight into the correct time at which the hazelnut should be sprayed to control the disease, and this could also be used to optimise the hazelnut production, supported by data regarding the latent infection period. Even if further data acquirement is strongly stressed and suggestions for research efforts are proposed, this is a good starting point for hazelnut value chain stakeholders.

## 4. Materials and Methods

**Figure 3:**
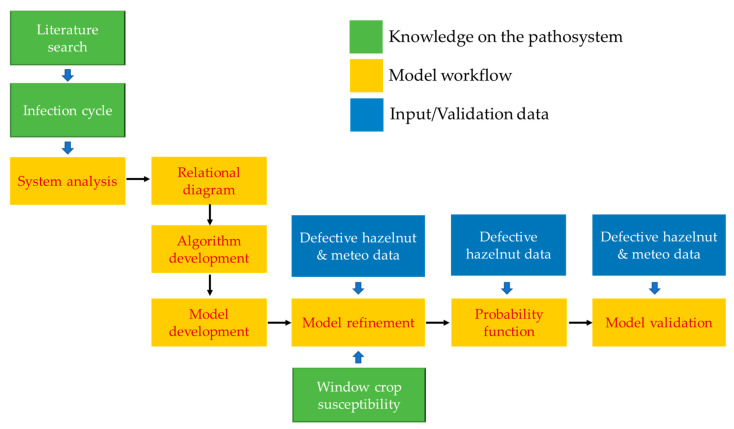
Workflow of study. Pathway for model development reported in yellow boxes (“Model workflow”), input data sources (DH and meteorological data) reported in blue boxes (“Input/Validation data”), and “Knowledge on the pathosystem”, referring to knowledge on *D. eres* infection cycle and hazelnut crop susceptibility, reported in green boxes.

We present the workflow of this study, expressed through several steps, in Figure 3. We use the different box colours to highlight the common parts, such as orange for the pathway of the model development, blue for the data input, and green for knowledge on the *D. eres* infection cycle and hazelnut susceptibility. We describe the workflow steps in the following paragraphs of this section.

### 4.1. System Analysis and Model Development

We considered all the quantitative data available in the literature regarding the *D. eres*–hazelnut pathosystem, as well as other *Diaporthe*-related pathosystems, to describe the infection cycle, with a focus on flower and fruit infection, and we drew the relational diagram.

#### 4.1.1. Infection Cycle

*D. eres* overwinter on twigs as pycnidial conidiomata [29], and their development is regulated by the air temperature (T) (°C) and water activity (*a_w_*) (0–1 scale). Mature pycnidia produce cirrhi, as well as plenty of α conidia, under suitable environmental conditions (temperature (T) (°C); relative humidity (RH) (%); rainfall (R) (mm)). Conidia are disseminated by R. The role of insect pests is unknown; therefore, we did not consider them in this study. Conidia reach female inflorescence (BBCH615, [44]) or young fruits from the setting (BBCH691) and germinate, tuned by the T and RH. Then, the mycelium grows and penetrates at the setting/fruit growing (BBCH751), nuts are infected, and later, the internal and external browning of the hazelnut becomes visible (Figure 1).

#### 4.1.2. Relation Diagram

We developed the relational diagram of *D. eres* on hazelnut (Figure 1) using the systems analysis syntax [25]. Briefly, the state variables (boxes) represent the pathogen stages in the infection cycle. The flow from one step to the next (arrows) is regulated by the rates (valves), which, in turn, are influenced by the external and auxiliary variables (short segments and circles, respectively). The external variables include weather variables, such as *T*, *RH*, *a*_*w*_, and *R*. Mathematical equations (dotted lines) link the rates to the external variables.

### 4.2. Algorithm and Model Development

#### Functions and Algorithm

The rate variables consist of mathematical equations taken from the literature, or those we developed using published data. The period of the crop susceptibility to infection is not known. Therefore, we calculated the model predictions both without limits in terms of the crop susceptibility and by setting different timeframes during which the primary infection can take place: (i) until the end of April (approximately BBCH691); (ii) until the end of May (BBCH710); (iii) until the end of June (BBCH755). We performed the data fitting and parameter estimation using the nonlinear regression procedure of SPSS (IBM SPSS Statistic 27, IBM Corp, Armonk, NY, USA), which uses the Levenberg–Marquardt algorithm to minimise the residual sums of the squares. We evaluated the goodness of fit using the standard error of the parameters and adjusted *R*^2^.

### 4.3. Model Input

We ran the model (Figure 1) using the hourly meteorological data as the input (T, RH, and R), recorded from 1 January to 31 December and provided by wireless weather stations (Vantage Pro2™, Davis Instruments, Hayward, CA, USA) placed near the hazelnut orchards. One weather station was established in Chitatskari for 2013–2016 [5], and ten stations were established in Turkey for 2017–2020 [6]. The model predictions accounted for the period from 1 January to the BBCH81 and BBCH89 growth stages.

### 4.4. Model Output

The DEFHAZ model produces the following outputs regarding the *D. eres* infection cycle: (i) the PydR(T): the daily production rate of pycnidia; (ii) the DisR: the day when the inoculum is dispersed; (iii) the GR(T): the growth rate; (iv) the INF: the cumulative infection; (v) the *Dh-I*: the cumulative infection index (final model output).

### 4.5. Field Data Collection

For the same orchards and years in which we collected the weather data, researchers have determined the incidence of defective hazelnut kernels in previous studies (44 orchards [4,5,6]). Briefly, we assessed the incidence of defective hazelnuts assessed through visual observations as the percentages of (i) visible defects (brown spots visible on the hazelnut kernel surface after shelling) and (ii) hidden defects (internal defects, visible after hazelnut kernel half-cutting), both of which contribute to the total defective hazelnuts (TDs). We assessed the defective hazelnut incidence at early ripening (BBCH81) and full ripening (BBCH89).

### 4.6. Model Refinement

#### 4.6.1. Window for Crop Susceptibility to Infection

We considered four different scenarios regarding the crop susceptibility to primary *D. eres* infection, as previously mentioned: (i) limited until the end of April (*Dh-I 30_04*); (ii) limited until the end of May (*Dh-I 30_05*); (iii) limited until the end of June (*Dh-I 30_06*); (iv) no limit for the infection. We collected all the *Dh-I* values provided by the model (*Dh-I NL*). Then, we performed a stepwise linear regression analysis to estimate the best timeframe for the fungal infection. We used the F-test as the criterium to include (F ≤ 0.05) or exclude (F ≥ 0.1) the independent variable.

#### 4.6.2. Probability Function

We developed a binary logistic regression (Equation (5)) using the hidden or total defective hazelnuts as the dependent variable. The independent variable used in the logistic equation was the *Dh-I* calculated in the timeframe selected by the stepwise regression:

(5) $$\begin{array}{c} P=\frac{1}{1+e^{c+b\times \left(risk\;index\right)}} \end{array}$$

This approach estimates the probability that an event will occur (0–1 scale: we consider that the event will occur when *P* > 0.5, and that it will not occur when *P* ≤ 0.5).

We considered the hidden or total defect incidence below/above a threshold, which we established as 1%. The independent variable used was the *Dh-I* produced by the model at the BBCH81 and BBCH89 growth stages. We used the logistic regression module of IBM SPSS Statistics (version 27.0) to estimate the logistic equation’s parameters (*b* and *c*).

#### 4.6.3. Model Internal Validation

We validated the model for its ability to predict the hidden and total defects (calculated as the sum of the visible and hidden defects) at both harvest times (BBCH81 and BBCH89). We compared the *Dh-I* to the data on the incidence of defective hazelnut kernels collected in the mentioned orchards. We used the same dataset mentioned in Section 4.6.2.

We prepared a contingency table (2 × 2) with the observed (O) and predicted (P) data to show the true positive proportion (TPP) or sensitivity (O+|P+), the true negative proportion (TNP) or specificity (O−|P−), the false positive proportion (FPP) (O−|P+), and the false negative proportion (FNP) (O+|P−).

## Figures and Tables

**Table 1:** Summary of functions used for fitting data collected in different steps of infection cycle and computed parameters (Arciuolo et al. [18], modified).

Rate	Variable	Function	Reference in Text	Parameter			
				*a*	*b*	*c*	*R* ^2^
PydR	GDD_*T*	Logistic	1	6.27	−0.01	0.96	0.950
PydR	GDD_*a_w_*	Logistic	1	9.64	−0.02	0.98	0.971
Ger_T_	*T*	Bete	2–3	13.49	1.10	3.69	0.793
Ger_RH_	*RH*	Polynomial	4	11.85	1110.80	* n.a.	0.975
GR_T_	*T*	Bete	2–3	5.06	1.39	3.46	0.964
GR_aw_	*a_w_*	Logistic	1	73.59	−79.05	0.97	0.987

* n.a.: not applicable.

**Table 2:** Parameter b and statistics of logistic regression (Equation (1)) applied to predict probability of having hidden and total defective hazelnuts (≥1%) as function of output index of predictive model in different considered scenarios.

	b	S.E.	Wald ^a^	df	Prob. ^b^	Exp (b) ^c^
**Hidden defects**						
BBCH81 vs. BBCH81						
*Dh-I 30-5*	0.000 ^d^	0.000	0.810	1	0.368	1.000
*Constant*	−2.449	0.621	15.533	1	0.000	0.086
BBCH89 vs. BBCH89						
*Dh-I 30-5*	0.000	0.000	1.340	1	0.247	1.000
*Constant*	−1.890	0.374	25.475	1	0.000	0.151
BBCH81 vs. BBCH89						
*Dh-I 30-5*	0.000	0.000	1.860	1	0.173	1.000
*Constant*	−1.908	0.366	27.244	1	0.000	0.148
**Total defects**						
BBCH81 vs. BBCH81						
*Dh-I 30-5*	0.000	0.000	3.914	1	0.048	1.000
*Constant*	−2.546	0.476	28.647	1	0.000	0.078
BBCH89 vs. BBCH89						
*Dh-I 30-5*	0.000	0.000	8.708	1	0.003	1.000
*Constant*	−1.033	0.297	12.127	1	0.000	0.356
BBCH81 vs. BBCH89						
*Dh-I 30-5*	0.000	0.000	5.861	1	0.015	1.000
*Constant*	−0.904	0.280	10.424	1	0.001	0.405

^a^ Wald statistic calculated for model variables to determine whether a variable should be removed; ^b^ probability level of parameter; ^c^ Exp(b): factor of increase in probability of event when independent variable changes by one unit; ^d^ decimal digits are available but not reported.

**Table 3:** Contingency matrix for model internal validation reported as model capability of correctly predicting defective hazelnuts (% of accurate predictions). A prediction is correct when the model prediction agrees with the observed data. We tested the model accuracy to predict hidden defective (HD) and total defective (TD) hazelnuts in three different comparison scenarios of predicted vs. observed: BBCH81 vs. BBCH81, BBCH89 vs. BBCH89, and BBCH81 vs. BBCH89. Grey cells indicate correct predictions for each scenario; white cells on left indicate underestimates (observed positive and predicted negative values); white cells on right suggest overestimates (observed negative and predicted positive values).

Hidden Defective Hazelnuts	Observed	Predicted	
		Negative	Positive
BBCH81 vs. BBCH81	Negative	95	0
	Positive	5	0
BBCH89 vs. BBCH89	Negative	84	0
	Positive	16	0
BBCH81 vs. BBCH89	Negative	84	0
	Positive	16	0
**Total Defective Hazelnuts**			
BBCH81 vs. BBCH81	Negative	89	0
	Positive	11	0
BBCH89 vs. BBCH89	Negative	56	5
	Positive	23	16
BBCH81 vs. BBCH89	Negative	59	3
	Positive	26	12

## Data Availability

Not applicable.
